# The impact of ACA Medicaid expansion on socioeconomic inequality in health care services utilization

**DOI:** 10.1371/journal.pone.0209935

**Published:** 2018-12-31

**Authors:** Shiho Kino, Ichiro Kawachi

**Affiliations:** Department of Social Behavioral Sciences, Harvard. T.H. Chan School of Public Health, Boston, Massachusetts, United States of America; University Complutense of Madrid, SPAIN

## Abstract

**Objective:**

We examined whether the Affordable Care Act (ACA) Medicaid expansion reduced socioeconomic inequalities in health care utilization.

**Methods:**

We used data from the Behavioral Risk Factor Surveillance System, covering the 50 U.S. states and the District of Columbia, between 2011 and 2016. We selected outcome indicators, viz. ability to afford needed health care, having a personal doctor, use of health services in the past year (routine check-up, flu shot and dental visits), and attending screenings for breast, cervical, and colon cancers. Socioeconomic status was measured by household income. We calculated two indices of inequality by household income for each outcome: Slope Index of Inequality (SII) and Relative Index of Inequality (RII). We estimated difference-in-differences models to examine the impact of ACA Medicaid expansion on socioeconomic inequality in use of health care services.

**Results:**

The ACA Medicaid expansion appeared to reduce the socioeconomic gap in individuals reporting financial ability in accessing health care (difference-in-differences estimators, -0.045 for SII and RII), having a personal doctor (-0.037 for SII and RII), and receiving routine check-ups (-0.027 for SII and -0.039 for RII). However, the expansion was not associated with reduction in the socioeconomic gap for preventive health care visits or dental care.

**Conclusions:**

The ACA Medicaid expansion had mixed effects on socioeconomic disparities in health care utilization. Medicaid expansion may not be sufficient to address socioeconomic disparities in preventive services uptake.

## Introduction

Health insurance coverage helps increase access to health care services including preventive and primary care and ultimately could contribute to population health improvement [1]. In 2010, as a part of the Affordable Care Act (ACA), Medicaid expansion was signed into law to improve access to health insurance for low-income Americans living below 138% of federal poverty level [2]. Originally the law regarding the Medicaid expansion was planned for the whole country, but in 2012 the U.S. Supreme Court ruled the individual states could opt out [3]. By September 2016, 32 states decided to expand Medicaid while other states opted not to [4]. This created a natural policy experiment to examine the impact of health insurance expansion on different health outcomes.

A growing number of studies have examined the impact of the ACA and a recent review concluded that the legislation succeeded in expanding insurance coverage as well as access to some types of care [5]. For example, the ACA expansion was associated with a reduction in the uninsured rate, while increasing the use of some health services, such as outpatient visits and overnight hospital stays, as well as the use of preventive services (e.g. screening visits and check-ups including cholesterol screening, Pap smear tests, mammograms and PSA tests) [6–9]. In addition, expansion states had a decrease in the prevalence of households reporting inability to afford needed follow-up care and worry about paying medical bills [6].

Historically, income-related inequality in access to healthcare has been substantially higher in the US compared to other developed countries [10]. Griffith, Evans and Bor addressed the effect of the ACA Medicaid expansion on socioeconomic disparities using the Behavioral Risk Factor Surveillance System (BRFSS) data and stratifying each socioeconomic group, and found that it primarily benefited low income groups by expanding their access to healthcare [11]. In particular, the ACA promoted an increase in primary care utilization among all racial and ethnic groups while decreasing the racial and ethnic disparities in health care access and utilization [12,13]. An analysis of BRFSS data in 2014 found that income disparities in breast and cervical cancer screenings as well as flu vaccinations decreased in the Medicaid expansion states, while increasing in non-expansion states [14]. Hence, an important outcome of ACA expansion may be to reduce income-based disparities in the use of health care services.

To the best of our knowledge, however, there have been no previous studies that directly addressed the impact of ACA Medicaid expansion on socioeconomic inequalities in healthcare services utilization.

## Methods

### Data source and study sample

The data were from the BRFSS between 2011 and 2016 in order to cover the periods before and after the ACA Medicaid expansion (launched in 2014). The BRFSS is an annual telephone survey conducted by the Centers for Disease Control and Prevention to monitor state trends in health-related risk behaviors, chronic health conditions, and use of preventive services [15]. Each survey includes more than 400,000 individuals aged 18 years and older residing in the 50 states, the District of Columbia and three U.S. territories. In this study, we excluded three U.S. territories and the individuals aged 65 years old and older because they were not targeted for the ACA Medicaid expansion.

### Outcome variables

We used 9 binary items related to the access to care and the use of different health services, namely, having a personal doctor, financial ability to access to care, having a routine check-up in the past year, having a flu vaccination (shot or spray) in the past year, having a dental visit in the past year, having a clinical breast exam, mammogram or Pap test in the past year, having a sigmoidoscopy/colonoscopy test in the past year. Most states did not ask about clinical breast exam, Pap test, mammography, sigmoidoscopy/colonoscopy or PSA tests in 2013 and 2015. Similarly, clinical breast exams were not asked in most states in 2016. Hence, we used information about clinical breast exams in 2012 and 2014 and information about Pap tests, mammograms, sigmoidoscopies/colonoscopies and PSA tests in 2012, 2014 and 2016. For all other outcomes, we used annual data available between 2011 and 2016. In addition, a Pap test and a clinical breast exam are recommended for women aged 21 and older [16]. A mammogram is recommended for women aged 50 and older and sigmoidoscopy/colonoscopy is recommended for those aged 50 and older [16]. The dataset did not allow us to make set a cutoff point at 21 years old, hence we used women aged 25 and older for access to Pap tests and clinical breast exams. Use of a mammogram (only for women) and sigmoidoscopy/colonoscopy was restricted to those aged 50 years and older.

### Calculation of SII and RII

We calculated two indices of socioeconomic inequality in health services utilization; the Slope Index of Inequality (SII) and the Relative Index of Inequality (RII) [17]. The SII is an absolute measure of socioeconomic disparity in health outcome. This measure reflects changes in the mean or the prevalence of the health outcome among the population. On the other hand, the RII measures the relative magnitude of inequality between socioeconomic groups as it is obtained from the normalized slope coefficient by the group mean. For example, when the same absolute amount of the health outcome increases in all socioeconomic groups, the SII would increase while the RII would not change.

In order to calculate the SII, social groups are ordered from lowest to highest. In this study, socioeconomic status was measured by income in five categories (i-v): (i) less than $15,000, (ii) $15,000 to less than $25,000, (iii) $25,000 to less than $35,000, (iv) $35,000, to less than $50,000 and (v) $50,000 or more. The population of each income-group category covers a range in the cumulative distribution of the population and is given a score based on the midpoint of its range in the cumulative distribution in the population [18]. Then, health service utilization is plotted against this midpoint variable, and a regression line is fitted.

The SII is obtained via regression of the mean outcome variables on the mean relative rank variable;

$${\overset{-}{y}}_{j}=\beta_{0}+\beta_{1}{\overset{-}{R}}_{j}$$

Where *j* represents income group, ${\overset{-}{y}}_{j}$ is the average health service utilization, ${\overset{-}{R}}_{j}$ is the average relative ranking of income group *j*, β_0_ is the estimated health service utilization of a hypothetical person at the bottom of the socioeconomic hierarchy, and β_1_ is the difference in average health service utilization between the hypothetical person at the bottom of the income group and the hypothetical person at the top. The coefficient β_1_ is the SII, which is interpreted as the absolute difference in health service utilization between the bottom and top of the income group distribution. The RII is calculated by dividing the SII by the mean of health service utilization among the whole population in each year in each state (μ); RII = SII / μ = β_1_ / μ.

### Statistical analysis

The SII and the RII were calculated for each state by year in a first step, and then the difference-in-differences analysis (DiD) was conducted at the state-level, with each state representing one observation. For the DiD, the treatment group was defined as the states which expanded Medicaid by December 2015 while the control group included the states which did not expand Medicaid or later than December 2015. Officially ACA expansion was implemented by January 2014 in most of the states, however, some states that expanded before January 2016 (Michigan expanded in April 2014, New Hampshire in August 2014, Pennsylvania in January 2015, Indiana in February 2015, and Alaska in September 2015) were also classified together with the expanded states, following the procedure described by Simon, Soni and Cawley [5]. Therefore, the DiD estimator compares the 31 expanded states with the 20 non-expansion states (The list is available on S1 Table). DiD is based on a parallel trend assumption prior to implementation of the intervention. Therefore, we conducted a pre-trend test for each outcome to examine the null hypothesis that there is no significant difference in the linear trends between expansion and non-expansion states. The study was exempt from human subjects approval (non-identifiable data; not human subjects).

STATA 14 [19] was used for all statistical analyses. Weighted percentages for demographic characteristics of the sample population were calculated. The BRFSS design weights were used to adjust for noncoverage and nonresponse and to force the total number of cases to approximate population estimates for each geographic region, controlling for age group by gender, race or ethnicity, education, marital status, tenure, gender by race or ethnicity, age group by race or ethnicity, and phone ownership [20].

Two DiD models were constructed using SII and RII in each year in each state for each outcome to compare before and after the ACA Medicaid expansion and to compare between the aggregated data before the ACA (2011–13) and the data from each of the years after the ACA implementation (2014–16). All analyses were controlled for state- and year- fixed effects.

## Sensitivity analysis

Depending on the U.S. state, the changes to Medicaid eligibility for adults were only mild to substantial [21]. We conducted a sensitivity analysis by repeating the DiD regression only comparing the full-expansion states to the non-expansion states, i.e. we excluded the substantial expansion states (California, Connecticut, Hawaii, Minnesota and Wisconsin) and the mild expansion states (Delaware, District of Columbia, Massachusetts, New York and Vermont).

In addition, we conducted another sensitivity analysis excluding the states expanding Medicaid eligibility only after January 2014 (Alaska, Indiana, Louisiana, Michigan, Montana, New Hampshire and Pennsylvania).

Furthermore, among the Medicaid expansion states, Montana and North Dakota did not provide the same dental benefits for adults as in other states. Therefore, we excluded those two states in additional sensitivity analysis examining the effects of ACA on dental visits.

## Results

Total sample size was 1,874,151. The response rate was slightly less than 50% every year for both landline and cell phones. Table 1 presents the weighted demographics of the sample population. Non-expansion states included more black people while expansion states had more Hispanic and multiracial people. The expansion states included higher income and more educated respondents compared to non-expansion states. In both groups of expansion and non-expansion states, income and education slightly increased during the period of the study.

**Table 1:** Demographic of the study sample.

	Expansion states		Non-expansion states	
	2011–13	2014–16	2011–13	2014–16
Sex				
Men	42.76	45.01	41.33	44.11
Women	57.24	54.99	58.67	55.89
Age				
18–24	7.48	8.18	7.58	8.57
25–34	14.72	14.62	15.08	15.63
35–44	18.85	17.61	18.36	18.01
45–54	26.55	25.24	25.85	24.12
55–64	32.40	34.34	33.12	33.67
Marital status				
Single	41.45	41.27	39.62	39.70
Married	58.55	58.73	60.38	60.30
Race				
White (non-Hispanic)	78.54	78.70	79.87	78.94
Black	6.95	6.72	10.32	10.09
Hispanic	7.48	7.40	5.13	6.28
Multiracial	2.13	2.21	1.48	1.47
Other	4.90	4.98	3.21	3.22
Income				
Less than $15,000	10.81	9.82	12.36	10.62
$15,000 to less than $25,000	14.15	13.48	16.53	15.78
$25,000 to less than $35,000	9.40	8.63	10.58	10.01
$35,000 to less than $50,000	13.41	12.59	14.80	14.16
$50,000 or more	52.23	55.49	45.73	49.43
Education				
Did not graduate High School	6.82	6.65	8.20	7.82
Graduated High School	26.27	25.90	28.36	28.09
Attend College/Technical School	27.57	27.30	29.43	29.32
Graduated from College/Technical School	39.34	40.16	34.01	34.77

**Fig 1:**
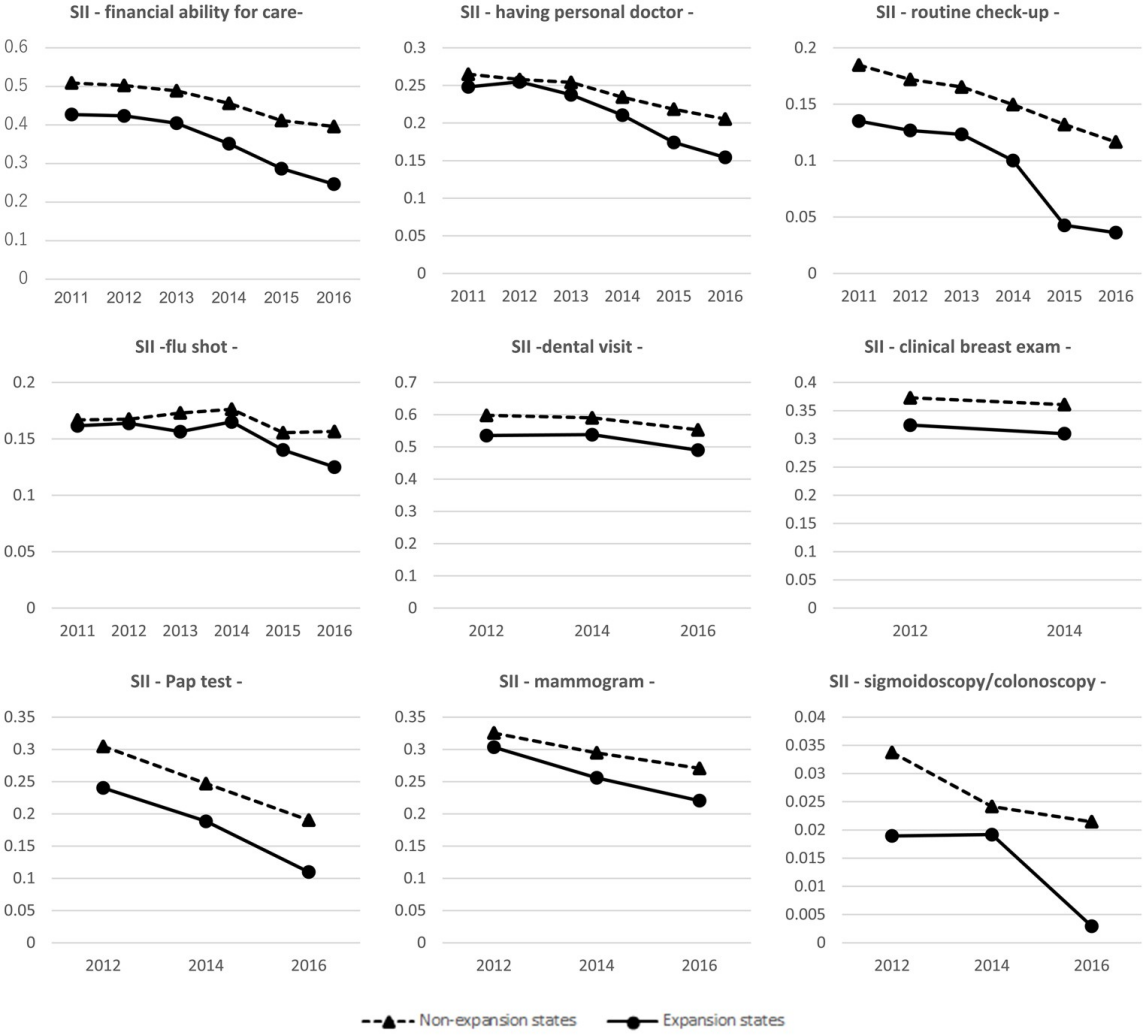
Trends in SII for expansion and non-expansion states.

**Fig 2:**
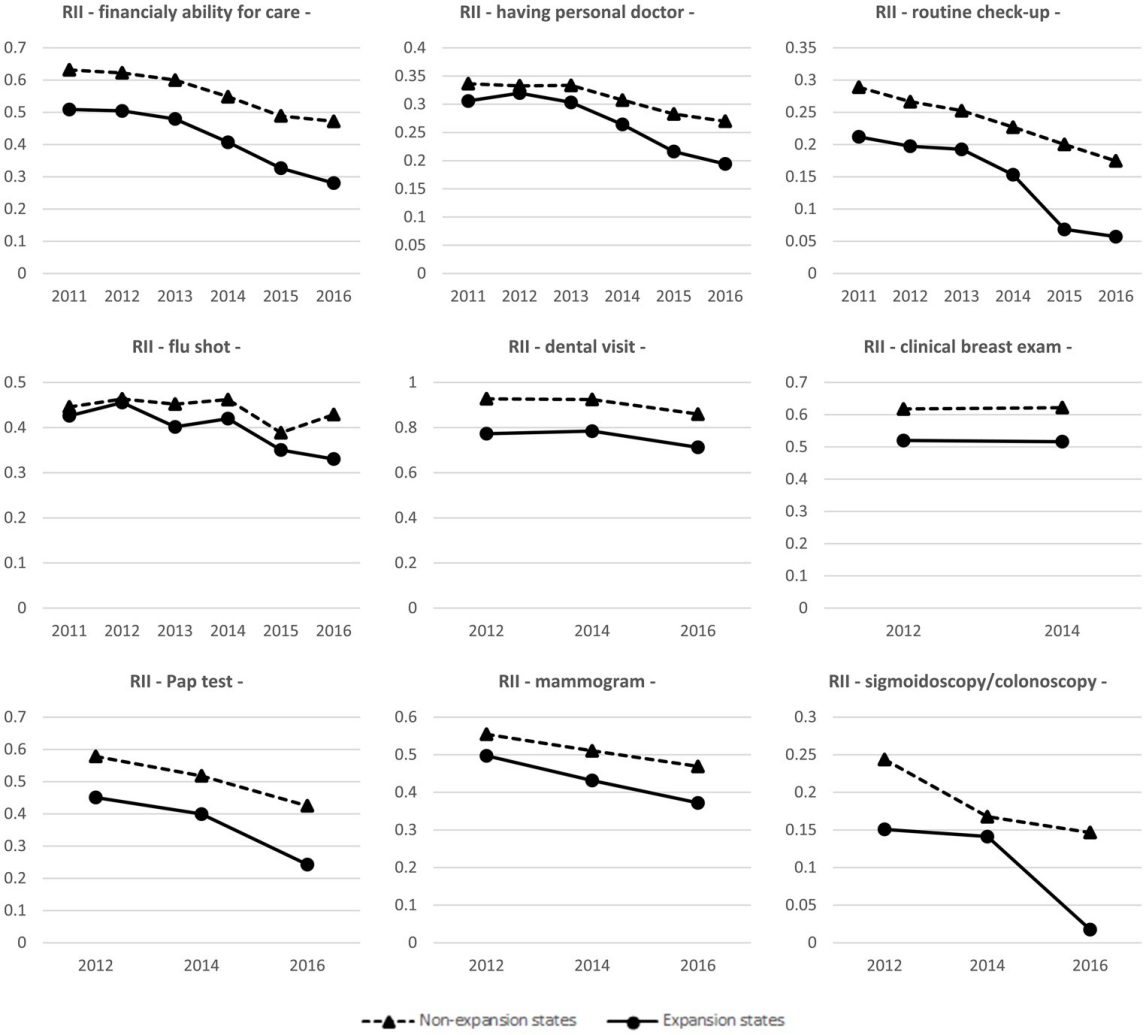
Trends in RII for expansion and non-expansion states.

Figs 1 and 2 present the trends in SII and RII for expansion and non-expansion states. In general, the expansion states exhibited lower socioeconomic disparities in health services utilization (both SII and RII) compared to the non-expansion states. Means of SII and RII for expansion and non-expansion states before and after the expansion are presented in S2 Table. We also conducted a pre-trend test for each outcome and found that the null hypothesis (that there is no statistically significant difference in the linear trends between expansion and non-expansion states) was not rejected for all outcomes, which supports a linear trend assumption in outcomes during the pre-implementation period in the expansion and non-expansion states.

Table 2 shows the results of difference-in-differences models. Income-based inequalities in individuals reporting financial ability to access to care decreased by 4.5% (coefficient: -0.045; 95%CI: -0.062, -0.027) compared to before the expansion with 4.3% and 6.8% reductions in 2015 (coefficient: -0.043; 95%CI: -0.068, -0.019) and 2016 (-0.068; 95%CI: -0.092, -0.043) respectively. Relative inequality in the same outcome (measured by the RII) also decreased by 4.5% (coefficient: -0.045; 95%CI: -0.068, -0.022) compared to before the expansion with 4.2% (-0.042; 95%CI: -0.074, -0.009) in 2015 and 7.1% in 2016 (-0.071; 95%CI: -0.104, -0.039). Similarly, income-based inequalities in having personal doctors and receiving routine check-ups also decreased by 2.8% (-0.028; 95%CI: -0.044, -0.011) and 2.8% (-0.028; 95%CI: -0.045, -0.010) respectively after the expansion. Relative inequality in both outcomes also decreased 3.7% (-0.037; 95%CI: -0.059, -0.015) and 3.9% (-0.039; 95%CI: -0.066, -0.012) respectively.

**Table 2:** Difference-in-Differences estimates of Medicaid expansion implementation in SII and RII for use of health services.

		Estimate (S.E.) 2014	Estimate (S.E.) 2015	Estimate (S.E.) 2016	Estimate (S.E.) 2014–2016
Financial ability	SII	-0.023	-0.043**	-0.068***	-0.045***
		(0.012)	(0.012)	(0.012)	(0.009)
	RII	-0.021	-0.042*	-0.071***	-0.045***
		(0.016)	(0.016)	(0.016)	(0.012)
Having personal doctor	SII	-0.012	-0.032**	-0.039**	-0.037**
		(0.012)	(0.012)	(0.012)	(0.011)
	RII	-0.019	-0.042**	-0.051**	-0.037**
		(0.016)	(0.016)	(0.016)	(0.011)
Routine check-up	SII	-0.004	-0.044***	-0.035**	-0.027**
		(0.012)	(0.012)	(0.012)	(0.009)
	RII	-0.005	-0.063**	0.049*	-0.039**
		(0.019)	(0.019)	(0.019)	(0.014)
Flu shot	SII	-0.003	-0.007	-0.023*	-0.011
		(0.012)	(0.012)	(0.012)	(0.008)
	RII	-0.016	-0.012	-0.073*	-0.034
		(0.030)	(0.030)	(0.030)	(0.022)
Dental visit	SII	0.010		-0.001	0.005
		(0.017)		(0.017)	(0.015)
	RII	0.015		0.008	0.011
		(0.027)		(0.027)	(0.024)
Clinical breast exam	SII	-0.003			-0.003
		(0.017)			(0.017)
	RII	-0.008			-0.008
		(0.030)			(0.030)
Pap test	SII	0.006		-0.016	-0.005
		(0.021)		(0.021)	(0.018)
	RII	0.009		-0.055	-0.023
		(0.045)		(0.045)	(0.039)
Mammogram	SII	-0.017		-0.028	-0.022
		(0.028)		(0.028)	(0.024)
	RII	-0.021		-0.040	-0.031
		(0.046)		(0.046)	(0.039)
Sigmoidoscopy/colonoscopy	SII	0.010		-0.004	0.003
		(0.017)		(0.017)	(0.015)
	RII	0.067		-0.036	0.015
		(0.111)		(0.111)	(0.096)

*p<0.05,

**p<0.01,

***p<0.001

All models were controlled for state- and year- fixed effects.

On the other hand, the SII and RII of other health outcomes were not materially affected by the ACA Medicaid expansion. Notably, the ACA Medicaid expansion was not associated with reduction of income-based inequalities in the utilization of preventive health care services including having visiting a dentist, getting a flu shot, or screening for breast, colon and cervical cancers.

The sensitivity analyses excluding substantial and mild expansion states made our findings more robust; we found that income-based inequalities in all non-preventive health care services (e.g. financial ability to access to care, having personal doctor and routine check-up) were significantly reduced (Table 3). Compared to before the expansion, inequality in financial ability to access doctors was reduced by 5.9% (95%CI; -0.078, -0.041) for SII and 6.7% (95%CI; -0.092, -0.042) for RII, inequality in having personal doctor decreased by 4.0% (95%CI; -0.058, -0.022) for SII and 5.4% (95%CI; -0.077, -0.030) for RII, and inequality in routine check-up redacted by 4.7% (95%CI; -0.065, -0.029) for SII and 7.1% (95%CI; -0.099, -0.042) for RII in the fully expanded states after the expansion.

**Table 3:** Difference-in-differences estimates of Medicaid expansion implementation in SII and RII for use of health services between full expansion and non-expansion (excluding substantial and mild expansion states).

		Estimate (S.E.) 2014	Estimate (S.E.) 2015	Estimate (S.E.) 2016	Estimate (S.E.) 2014–2016
Financial ability	SII	-0.027*	-0.059***	-0.092***	-0.059***
		(0.013)	(0.013)	(0.013)	(0.010)
	RII	-0.030	-0.066***	-0.105***	-0.067***
		(0.017)	(0.017)	(0.017)	(0.013)
Having personal doctor	SII	-0.021	-0.051***	-0.049***	-0.040***
		(0.013)	(0.013)	(0.013)	(0.009)
	RII	-0.030	-0.066***	-0.065***	-0.054***
		(0.017)	(0.017)	(0.017)	(0.012)
Routine check-up	SII	-0.016	-0.066***	-0.060***	-0.047***
		(0.013)	(0.013)	(0.013)	(0.009)
	RII	-0.024	-0.098***	-0.090***	-0.071***
		(0.020)	(0.020)	(0.020)	(0.015)
Flu shot	SII	0.003	-0.007	-0.015	-0.006
		(0.013)	(0.013)	(0.013)	(0.009)
	RII	-0.004	-0.018	-0.058	-0.027
		(0.034)	(0.034)	(0.034)	(0.021)
Dental visit	SII	0.001		-0.010	-0.004
		(0.017)		(0.017)	(0.032)
	RII	-0.002		-0.012	-0.007
		(0.028)		(0.028)	(0.024)
Clinical breast exam	SII	-0.004			-0.004
		(0.018)			(0.018)
	RII	-0.009			-0.009
		(0.032)			(0.032)
Pap test	SII	0.007		-0.035	-0.014
		(0.023)		(0.023)	(0.020)
	RII	0.016		-0.091	-0.038
		(0.050)		(0.050)	(0.044)
Mammogram	SII	-0.032		-0.055	-0.044*
		(0.024)		(0.024)	(0.020)
	RII	-0.050		-0.086*	-0.068
		(0.040)		(0.040)	(0.034)
Sigmoidoscopy/colonoscopy	SII	-0.003		-0.022	-0.013
		(0.016)		(0.016)	(0.014)
	RII	-0.013		-0.136	-0.074
		(0.113)		(0.113)	(0.098)

*p<0.05,

**p<0.01,

***p<0.001

All models were controlled for state- and year- fixed effects.

Our next sensitivity analysis excluding the states expanding later than January 2014 also found similar results as in the main analysis; the SII of financial ability to access care (coefficient: -0.048; 95%CI: -0.067, -0.029), having a personal doctor (-0.032; 95%CI: -0.050, -0.014), and a routine check-up (-0.031; 95%CI: -0.048, -0.013) were significantly reduced. The RII of these outcomes were also significantly decreased.

Lastly excluding the two states that did not include dental visits in the Medicaid expansion did not alter our main findings.

## Discussion

Our study focused on the impact of ACA Medicaid expansion on socioeconomic inequalities in the use of health services. The ACA Medicaid expansion appeared to mitigate income-based inequality related to difficulties in accessing health services due to financial reasons. On the other hand, we did not detect a significant impact of the expansion in reducing pre-existing inequalities in utilization of preventive services such as screening visits, and dental visits.

Our findings corroborate the results of an analysis of the National Health Interview Survey, which revealed that Medicaid expansion was related to a decrease in the rate of inability to afford needed follow-up care and worry about paying bills [6]. Our findings are also in line with a study that examined the impact of ACA implementation in Kentucky, which found that Medicaid expansion was related to a decrease in the uninsured population and in unmet medical needs due to cost, especially among high-poverty communities, while physician visitation in the past year remained unaffected [22]. Finally, Choi, Lee and Matejkowski [9] used the BRFSS to identify that the ACA expansion was associated with a range of improved outcomes, including health insurance coverage, having a personal doctor, and having a routine check-up in the past year.

Some previous studies reported that Medicaid expansion was associated with an increase in use of preventive health services including cervical, breast and prostate cancer screenings among socioeconomically disadvantaged people [7,8]. However, these studies did not explicitly address changes in socioeconomic disparity in the use of preventive services. That is, Medicaid expansion might help to improve access to preventive services for disadvantaged people, but it may not improve the uptake of the same services to a sufficient degree to offset pre-existing inequalities. For example, a study looking at the effect of the Massachusetts Health Insurance Reform found that improved health insurance coverage was not associated with an increase in use of screenings such as mammography [23].

Taken together, our results imply that ACA Medicaid expansion strengthens the safety net for the poor, but may not be sufficient—by itself—to affect income-based disparities in utilization of preventive health care services. The reason is because improved access does not necessarily translate to improved utilization, especially for preventive services. There are additional barriers faced by low-income people besides financial costs—e.g. lack of flexibility in work schedules to make dental appointments, lack of knowledge, inability to arrange childcare to receive screening services [24–26]. It might be because attendance of health services needs not only treatment fee but also travelling cost and time and taking time off from day jobs [27]. Thus, reducing socioeconomic inequality in use of health services needs not only providing health insurance, but also needs different strategies to approach the inequality.

In the case of emergency care, access is more tightly linked to utilization. Indeed a previous study found that emergency department visits increased in the wake of Medicaid expansion [28,29]. An increase in emergency department visits by the poor is not necessarily a desirable outcome, given that the emergency department use by uninsured patients can be due to being unable to obtain primacy care [30]. In fact, Sommers et al. also found that ACA expansion was related to a decrease in use of emergency departments, accompanied by a parallel increase in outpatient visits [8].

There have been previous natural policy experiments on the impacts of expanded health insurance coverage on socioeconomic health disparities. The largest one its kind is perhaps related to the establishment of the National Health Service (NHS) in Great Britain after the Second World War. Politicians of the time eagerly anticipated that the establishment of the NHS would abolish socioeconomic inequalities in health by freeing the people from the misfortune of illness. Unfortunately, their optimism turned out to be misplaced, because we now understand that much of illness and premature mortality is ascribable to the social determinants of health, i.e. to factors operating outside the realms of health insurance and health care utilization [31].

Indeed socioeconomic gaps in premature mortality have continued its steady upward march in Great Britain, in tandem with that nation’s growing inequalities in socioeconomic circumstances [32]. Our findings support the statement by Shaw, Smith and Dorling Shaw [33] that raising the living standards of some of the poorest people has not contributed to a reduction in overall inequalities in health. Thus, tackling socioeconomic inequalities in health need to approach not only access to insurance but also other determinants of health.

Some limitations of our study should be noted. The data were from a telephone interview survey, which meant that the poorest segments of the population may have been excluded. In addition, the response rate is low (slightly less than 50%) although it is significantly higher compared to other telephone surveys. We used BRFSS survey weights to improve the representativeness of the population. The ACA expansion was initiated since 2014 and the available data were until 2016, which might not be a sufficient interval to document the impact of ACA Medicaid expansion since changing behaviors would take time.

In conclusion, the ACA expansion might reduce socioeconomic inequality in inability to afford health care services, having a personal doctor, and attending a routine check-up, which are important to play a role as a safety net. On the other hand, the expansion might not contribute to a reduction in socioeconomic inequality in use of preventive attendance such as cancer screenings. Therefore, the specific strategies for reducing inequality would be needed.

## Supporting information

S1 Table Classification of expansion and non-expansion states. (DOCX)

S2 Table Mean Slope Index of Inequality (SII) and mean Relative Index of Inequality (RII) for expansion and non-expansion states before and after ACA expansion. (DOCX)
