# Supplementary material for: The impact of ACA Medicaid expansion on socioeconomic inequality in health care services utilization
Source: PLoS One. 2018 Dec 31;13(12):e0209935. doi: 10.1371/journal.pone.0209935 (PMC6312270; doi:10.1371/journal.pone.0209935)
Supplement: S1 Table — (DOCX) [file pone.0209935.s001.docx]

| **Expansion states (N=31)** | **Non-expansion states (N=20)** |
| --- | --- |
| ***Full expansion (n=21)*** | Alabama |
| Alaska | Florida |
| Arizona | Georgia |
| Arkansas | Idaho |
| Colorado | Kansas |
| Illinois | Louisiana |
| Indiana | Maine |
| Iowa | Mississippi |
| Kentucky | Missouri |
| Maryland | Montana |
| Michigan | Nebraska |
| Nevada | North Carolina |
| New Hampshire | Oklahoma |
| New Jersey | South Carolina |
| New Mexico | South Dakota |
| North Dakota | Tennessee |
| Ohio | Texas |
| Oregon | Utah |
| Pennsylvania | Virginia |
| Rhode Island | Wyoming |
| Washington |  |
| West Virginia |  |
| ***Substantial expansion (n=5)*** |  |
| California |  |
| Connecticut |  |
| Hawaii |  |
| Minnesota |  |
| Wisconsin |  |
| ***Mild expansion (n=5)*** |  |
| Delaware |  |
| District of Columbia |  |
| Massachusetts |  |
| New York |  |
| Vermont |  |
