# Supplementary material for: The impact of ACA Medicaid expansion on socioeconomic inequality in health care services utilization
Source: PLoS One. 2018 Dec 31;13(12):e0209935. doi: 10.1371/journal.pone.0209935 (PMC6312270; doi:10.1371/journal.pone.0209935)
Supplement: S2 Table — (DOCX) [file pone.0209935.s002.docx]

|  |  | **Expansion states** | | **Non-expansion states** | |
| --- | --- | --- | --- | --- | --- |
|  |  | **2011-2013** | **2014-2016** | **2011-2013** | **2014-2016** |
| Financial ability | SII | 0.418 | 0.295 | 0.500 | 0.421 |
|  | RII | 0.498 | 0.338 | 0.618 | 0.503 |
| Having personal doctor | SII | 0.247 | 0.180 | 0.259 | 0.219 |
|  | RII | 0.310 | 0.225 | 0.334 | 0.287 |
| Routine check-up | SII | 0.128 | 0.060 | 0.174 | 0.133 |
|  | RII | 0.201 | 0.093 | 0.269 | 0.201 |
| Flu shot | SII | 0.161 | 0.143 | 0.169 | 0.163 |
|  | RII | 0.428 | 0.367 | 0.454 | 0.427 |
| Dental visit | SII | 0.535 | 0.514 | 0.598 | 0.572 |
|  | RII | 0.773 | 0.749 | 0.928 | 0.892 |
| Clinical breast exam | SII | 0.325 | 0.309 | 0.373 | 0.361 |
|  | RII | 0.520 | 0.516 | 0.618 | 0.622 |
| Pap test | SII | 0.240 | 0.149 | 0.305 | 0.219 |
|  | RII | 0.451 | 0.321 | 0.578 | 0.471 |
| Mammogram | SII | 0.303 | 0.238 | 0.325 | 0.283 |
|  | RII | 0.497 | 0.402 | 0.554 | 0.490 |
| Sigmoidoscopy/colonoscopy | SII | 0.019 | 0.011 | 0.034 | 0.023 |
|  | RII | 0.151 | 0.079 | 0.244 | 0.157 |
